# The role and mechanism of the gut microbiota in the development and treatment of diabetic kidney disease

**DOI:** 10.3389/fphys.2023.1166685

**Published:** 2023-04-21

**Authors:** Xiaofang Wu, Lei Zhao, Yujiang Zhang, Kailong Li, Jurong Yang

**Affiliations:** ^1^ Department of Nephrology, The Third Affiliated Hospital of Chongqing Medical University, Chongqing, China; ^2^ Department of Nephrology, Chongqing Jiangjin Second People’s Hospital, Chongqing, China

**Keywords:** gut microbiota, diabetic kidney disease, inflammation, immunity, oxidative stress, gut microbiota-targeted therapies

## Abstract

Diabetic kidney disease (DKD) is a common complication in patients with diabetes mellitus (DM). Increasing evidence suggested that the gut microbiota participates in the progression of DKD, which is involved in insulin resistance, renin-angiotensin system (RAS) activation, oxidative stress, inflammation and immunity. Gut microbiota-targeted therapies including dietary fiber, supplementation with probiotics or prebiotics, fecal microbiota transplantation and diabetic agents that modulate the gut microbiota, such as metformin, glucagon-like peptide-1 (GLP-1) receptor agonists, dipeptidyl peptidase-4 (DPP-4) inhibitors, and sodium-glucose transporter-2 (SGLT-2) inhibitors. In this review, we summarize the most important findings about the role of the gut microbiota in the pathogenesis of DKD and the application of gut microbiota-targeted therapies.

## 1 Introduction

Diabetic kidney disease (DKD) is a major diabetic chronic microvascular complication in patients with diabetes mellitus (DM), characterized by clinical features of kidney function loss and albuminuria (Chen W. et al., 2021; Lin et al., 2018; Selby and Taal, 2020; Yang et al., 2021). Histological manifestations of DKD not only include main glomerular changes, such as thickening of the glomerular basement membrane, mesangial expansion, podocyte effacement and glomerular sclerosis, but also present as interstitial fibrosis, tubular atrophy, arteriolar hyalinosis and arteriosclerosis (Adeva-Andany et al., 2022; Lin et al., 2022; Selby and Taal, 2020). Around 30%–40% of all diabetic patients can develop DKD, which is the main cause of end stage renal disease (ESRD) worldwide and the major cause of morbidity and mortality in patients with DM (Fang Q. et al., 2021; Fernandes et al., 2019; Lin et al., 2018; Mosterd et al., 2021; Yang et al., 2021). The incidence of DKD has increased significantly over the past decade, and it continues to increase as our modern lifestyle changes towards inactivity habits, high-fat and high-fructose diet, gradually inducing obesity and insulin resistance (Fernandes et al., 2019; Yang et al., 2021). Long-time obesity and insulin resistance are the main factors responsible for type 2 DM(T2DM) development (Ashrafizadeh et al., 2022; Lv et al., 2022).

The pathogenesis of DKD is complex and remains not entirely clear, therefore, the management of DKD is not specific currently (Chen W. et al., 2021). Understanding the key pathogenesis allows identification of targeted treatment for DKD patients. Previous studies have identified that many pathways and mediators are involved in the development of DKD (Fang Q. et al., 2021; Lin et al., 2018; Samsu, 2021; Zhang et al., 2022). Renal hemodynamics changes, overactive of the renin-angiotensin-aldosterone system (RAAS), oxidative stress, and inflammation processes are the major established mechanisms involved in the pathogenesis of DKD, and renal fibrosis is the final common pathway (Fang Q. et al., 2021; Lin et al., 2018; Samsu, 2021; Zhang et al., 2022). Hyperglycemia causes afferent arteriolar dilatation by release of vasoactive mediators, and high local level of angiotensin II causes the constriction of efferent arteriole, resulting in glomerular hypertension and renal damage (Lin et al., 2018). The activation of RAAS is closely related to the change of intraglomerular hemodynamics and structure of the glomerulus and tubulointerstitium (Samsu, 2021). Oxidative stress has been established as a central factor in onset and progression of DKD, which causes damage to kidney cells and activate other immunological pathways, such as inflammatory and immunity, leading to increased proteinuria, accelerated tubulointerstitial fibrosis and renal failure (Kashihara et al., 2010; Samsu, 2021). Intensive glycemic and hypertension control, and RAAS inhibition have been demonstrated to delay disease progression in DKD (Chen W. et al., 2021; Li et al., 2020). Researchers are trying to explore other relevant factors. Accumulating evidence has indicated that the gut microbiota participates in the progression of DKD (Yang et al., 2021; Zhang et al., 2022). In this review, we summarize the most important findings regarding the role of the gut microbiota in DKD and describes how the gut microbiota affects the progression of DKD and the gut microbiota-targeted therapies.

## 2 The gut-kidney axis in the pathogenesis of DKD

The microbiota in healthy human intestines is a complex community of more than 100 trillion microbial cells with more than 1,000 different species (Chen et al., 2019; Nagase et al., 2022). In healthy condition, the gut microbiota lives in a symbiotic relationship with the host (Chen et al., 2019). However, the alterations of the normal composition of gut microbiota, known as gut dysbiosis, break the balance, resulting in various disease condition (Iatcu et al., 2021; Karalliedde and Gnudi, 2016; Li and Tang, 2018; Mosterd et al., 2021). In the case of DM, hyperglycemia promotes gut microbiota dysbiosis, which contributes to the development of DKD (Fernandes et al., 2019; Iatcu et al., 2021; Mosterd et al., 2021; Nagase et al., 2022; Zhao et al., 2018).

**FIGURE 1:**
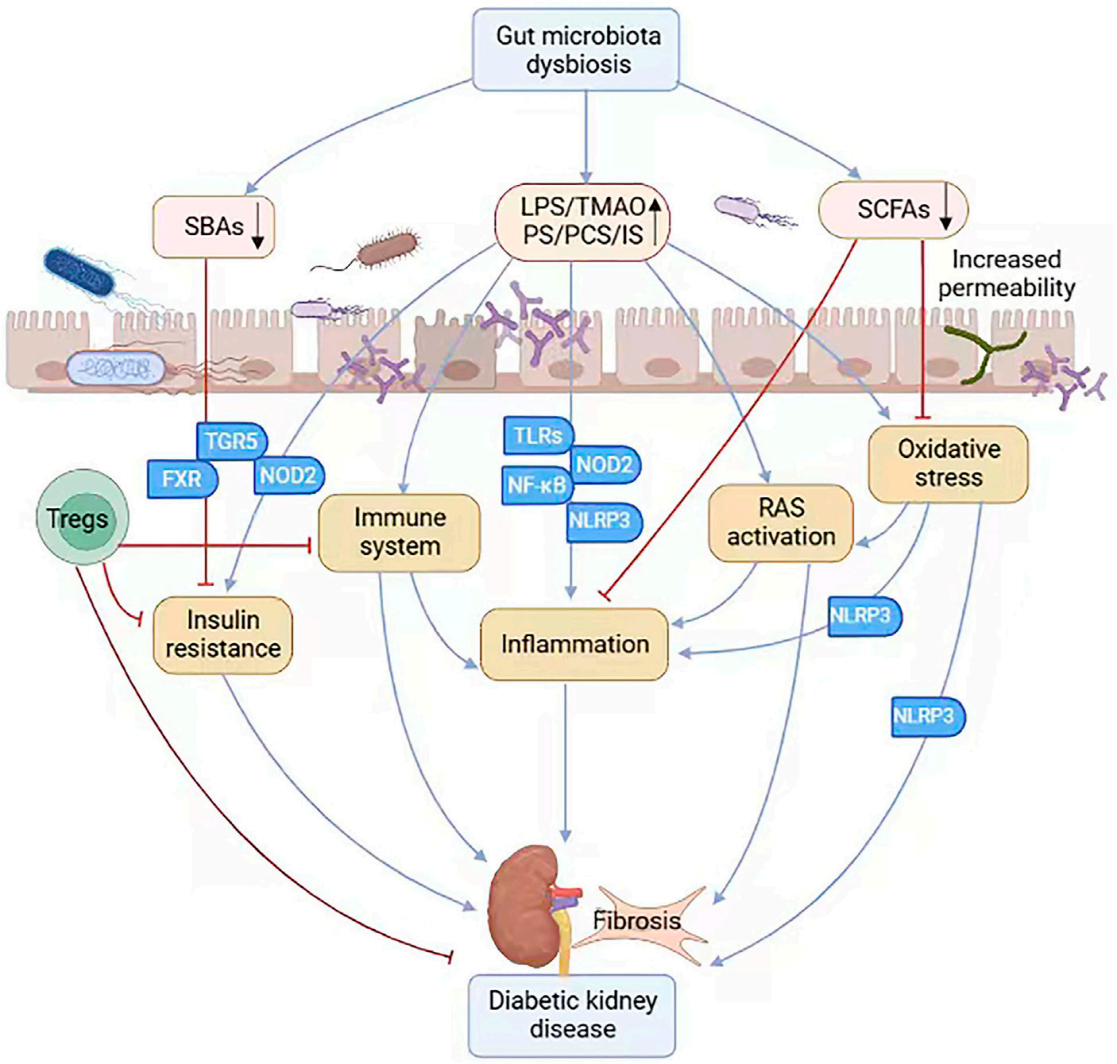
The gut-kidney axis in the pathogenesis of DKD. In patients with diabetes, increased uremic toxins induce gut microbiota dysbiosis, which increases the permeability of the epithelium and leads to increased exposure to uremic toxins and decreased production of SCFAs. Gut microbiota dysbiosis also induces deficiency of SBAs, which acts by binding to its receptor FXR and TGR5. The gut microbiota dysbiosis is involved in insulin resistance, RAS activation, oxidative stress, inflammation, immune system and fibrosis, contributing to the progression of diabetic kidney disease. (SCFAs, short-chain fatty acids; SBAs, secondary bile acids; FXR, nuclear farnesoid X receptor; TGR5, Takeda G protein-coupled receptor 5; RAS, renal renin-angiotensin system; Tregs, regulatory T cells; TLRs, Toll-like receptors; NOD2, nucleotide-binding oligomerization domain 2; NLRP3, nucleotide binding and oligomerization domain-like receptor family pyrin domain-containing 3).

The gut-kidney axis is defined to describe the relationship between gut microbiota and kidney diseases, including DKD (Chen et al., 2019; Evenepoel et al., 2017; Ni et al., 2022) (Figure 1). The interaction is bidirectional. On the one hand, increased uremic toxins affect the composition and function of gut microbiota in DKD. On the other hand, gut dysbiosis disrupts the intestinal barrier, increases the permeability of the epithelium, and leads to increased exposure to endotoxins, resulting in a series of adverse reactions and ultimately exacerbating kidney damage (Cai et al., 2020; Evenepoel et al., 2017; Ni et al., 2022; Wang P. et al., 2021; Zhao et al., 2019). Apart from renal failure itself, dietary restrictions, medication intake, and impaired gastrointestinal functions contribute to the alteration (Yang et al., 2021). Microbial metabolism shifts towards an increase of proteolytic microbes and excessive production of toxic metabolites, such as indoxyl sulfate (IS), p-cresol sulfate (PCS), phenyl sulfate (PS), trimethylamine N-oxide (TMAO), while a decrease of saccharolytic microbes mainly producing short-chain fatty acids (SCFAs), especially butyrate-producing bacteria (Evenepoel et al., 2017; Fernandes et al., 2019; Lin et al., 2022; Sabatino et al., 2017; Yang et al., 2021).

SCFAs, including acetate, propionate, and butyrate, are the primary end products of gut bacterial fermentation and play a key role in host colonic physiology, serving as a major source of energy for intestinal and colon cells, stimulating epithelial cell proliferation, and promoting glucose homeostasis (Cheng et al., 2022; Chi et al., 2021; Fernandes et al., 2019; Tanase et al., 2020). SCFAs mainly function through activating G-protein coupled receptors, like GPR41, GPR43, and GPR109A, and inhibiting histone deacetylase (HDAC) (Andrade-Oliveira et al., 2015; Cheng et al., 2022; Fang Q. et al., 2021; Huang et al., 2017b; Lin et al., 2022; Rooks and Garrett, 2016). In patients with DKD, the activation of GPRs by SCFAs stimulate the production of glucagon-like peptide-1 (GLP-1), improving blood glucose tolerance and insulin sensitivity (Deng L. et al., 2022; Fang Q. et al., 2021; Kim et al., 2014; Lin et al., 2022; Wang Y. et al., 2022). In regulating intestinal inflammation, SCFAs exert anti-inflammatory effects through increasing the expression of anti-inflammatory cytokine interleukin-10 (IL-10) and suppressing the production of inflammatory cytokines (IL-6 and TNF-α) and the activation of nuclear factor-κB (NF-κB) (Kim et al., 2014). In addition, sodium butyrate has also been found to exert protective effects on DKD rats through activation of autophagy (Cheng et al., 2022).

Considering all of the effects of intestinal microbiota, modulation of the gut-kidney axis may be a promising therapeutic target for controlling the progression of DKD.

## 3 Gut microbiota dysbiosis affects the progression of DKD

### 3.1 Gut microbiota dysbiosis and insulin resistance in DKD

Insulin resistance is recognized as the basis of histological and clinical manifestations of DKD (Adeva-Andany et al., 2022; Karalliedde and Gnudi, 2016; Penno et al., 2021). Previous studies have discovered that kidney structural changes of DKD usually precede the clinical diagnosis of type 2 diabetes (caused by insulin resistance) (Adeva-Andany et al., 2022; Akhtar et al., 2020). And in newly diagnosed patients with type 1 diabetes (caused by inadequate insulin production but not insulin resistance), morphological changes of DKD were absent, but appear after insulin resistance develops (Adeva-Andany et al., 2022).

Previous studies have shown that gut microbiota dysbiosis may lead to an increase of lipopolysaccharide (LPS)-producing microbiota in the gut, causing systemic mild inflammation and contributing to apoptosis of islet cells and insulin resistance in people with DM (Chi et al., 2021; Deng L. et al., 2022; Hu et al., 2020; Lin et al., 2022; Ni et al., 2022; Zhao et al., 2018). LPS receptors have been found to be key mediators in activating insulin resistance (Yoo et al., 2020). Increased level of toxic metabolites PCS and IS could also lead to insulin resistance (Koppe et al., 2013; Ni et al., 2022). Bile acids (BAs) are also metabolites derived from gut microbiota. Primary BAs are transformed into secondary bile acids (SBAs) by the gut bacteria, the latter could regulate glucose metabolism and alleviate insulin resistance and improve DKD by binding to its receptor the nuclear farnesoid X receptor (FXR) and the membrane-bound Takeda G protein-coupled receptor 5 (TGR5) (Deng L. et al., 2022; Fang Q. et al., 2021; Gao et al., 2022; Ni et al., 2022; Tanase et al., 2020; Wang Y. et al., 2022). The activation of the intestinal FXR plays an important role in the connection between BAs and gut microbiota (Fang et al., 2015; Gao et al., 2022; Han et al., 2021; Wang Y. et al., 2022). Gut microbiota dysbiosis induces SBAs deficiency and inhibits the activation of BAs receptors FXR and TGR5, further promoting inflammation and insulin resistance (Duan et al., 2021; Sinha et al., 2020).

In summary, intestinal microbiota dysbiosis is involved in insulin resistance and participates in the progression of DKD (Figure 1).

### 3.2 Gut microbiota dysbiosis and RAS activation in DKD

It is recognized that the activation of local renal renin-angiotensin system (RAS), rather than circulating RAS, is one of the main initiators of DKD (Chi et al., 2021; Lu et al., 2020; Lu et al., 2018; Tanase et al., 2020; Wysocki et al., 2017). Uremic toxins and hyperglycemia could promote the production of angiotensin II (Ang-II), the most important components of RAS, which in turn induces renal vasoconstriction and glomerular hyperfiltration, inflammatory and profibrotic factors secretion, extracellular matrix deposition, as well as morphological changes of podocytes, accelerating the progression of DKD (Chi et al., 2021; Lu et al., 2020; Lu et al., 2018; Tanase et al., 2020).

Accumulating evidence suggests an interaction between gut microbiota and RAS activation (Chi et al., 2021; Jaworska et al., 2021; Lu et al., 2020). The fermentation of gut microbiota produces SCFAs, which may exert protective or causative effects on DKD (Fang Q. et al., 2021; Huang et al., 2020). Although much evidence suggests SCFAs exert protective effects in DKD (Fang Q. et al., 2021; Li et al., 2020; Ni et al., 2022; Yang et al., 2021), recent studies have indicated that the dysbiosis of gut microbiota may produce excessive SCFAs, especially acetate, which could bind to receptors in the kidney and regulate intrarenal RAS, thus exerting pathological changes in early DKD (Chi et al., 2021; Hu et al., 2020; Lu et al., 2020; Lu et al., 2018). The local gastrointestinal RAS is also regarded as a potential mediator of microbiota-related effects in DKD. The gut bacteria could influence the process mediated by gastrointestinal RAS in intestinal, such as glycemic homeostasis and inflammatory process (Jaworska et al., 2021). In addition, the intestinal microbiota and its metabolites can transfer through the intestinal epithelium with increased permeability, stimulating macrophages to phagocytose bacteria and release inflammatory factors, thus causing damage to endothelial cells and podocytes of kidney through activation of the RAS system and inflammation process (Andersen et al., 2017; Chi et al., 2021; Lu et al., 2020; Tanase et al., 2020; Wang P. et al., 2021).

Collectively, these studies suggest that there might be a causal relationship between gut microbiota dysbiosis and RAS activation in DKD (Figure 1). Therapeutic interventions to alter gut microbiota and inhibit RAS activation may be applied to ameliorate the kidney damage.

### 3.3 Gut microbiota dysbiosis and inflammation and immunity in DKD

Although DKD is traditionally considered as a non-immune disease, a growing body of research suggest that inflammatory responses and immune system may play a major role in the pathogenesis of DKD (Donate-Correa et al., 2015; Du et al., 2013; Fernandes et al., 2019; Li et al., 2020; Samsu, 2021). The innate immune activation and inflammation have been found to be associated with insulin resistance and DKD (Du et al., 2013; Tang and Yiu, 2020; Wada and Makino, 2016).

The innate immune system includes a large family of pattern recognition receptors (PRRs), including membrane-bound Toll-like receptors (TLRs) and nucleotide-binding oligomerization domain (NOD)-like receptors (NLRs), which recognize pathogen-associated molecular patterns (PAMPs) and danger-associated molecular patterns (DAMPs) and initiate the proinflammatory cascade (Du et al., 2013; Fernandes et al., 2019; Wada and Makino, 2016). The TLRs family member, especially TLR2 and TLR4, have been demonstrated to activate inflammatory process, such as NF-κB signaling pathway, and play a critical role in promoting tubular inflammation in DKD (Du et al., 2013; Gluba et al., 2010; Lin et al., 2013; Lin et al., 2012; Mosterd et al., 2021; Mudaliar et al., 2014; Tang and Yiu, 2020). Activation of the NOD-like receptors (NLRs) family members, especially NOD2 and nucleotide binding and oligomerization domain-like receptor family pyrin domain-containing 3 (NLRP3), have also been proved to exert a crucial effect on the progression of DKD (Du et al., 2013; Fernandes et al., 2019; Lv et al., 2022). NOD2 could participate in hyperglycemia-induced podocyte dysfunction and mediate inflammation and insulin resistance in diabetic nephropathy (Du et al., 2013; Fernandes et al., 2019). And the NLRP3 inflammasome activation has also been reported to induce pro-inflammatory cascades via the induction of IL-1β and IL-18 and perpetuate inflammation in DKD (Chen et al., 2013; Mosterd et al., 2021; Shahzad et al., 2015; Tang and Yiu, 2020; Wu et al., 2018).

Growing evidence suggests that the innate immune complement system participate in the development of DKD (Flyvbjerg, 2017; Li et al., 2021; Sun et al., 2019; Tan et al., 2022; Yiu et al., 2018). Hyperglycemia leads to increased complement regulatory protein glycation and increased activation of the complement cascade through mannan-binding lectin (MBL) pathway due to accelerated protein glycation (Tan et al., 2022; Tang and Yiu, 2020). Increased complement activation promotes the production of complement end products anaphylatoxins C3a and C5a, which activate G-protein-coupled receptors, C3aR and C5aR1, leading to overproduction of reactive oxygen species (ROS) and inflammatory cytokines, thereby resulting in inflammation in DKD (Tan et al., 2022; Wang P. et al., 2021). The NLRP3 inflammasome is also involved in the mechanism of complement-mediated renal damage in DM (Tan et al., 2022).

In addition, regulatory T cells (Tregs) are CD4^+^ T helper (Th) cells that suppress immune and inflammatory responses (Ghali et al., 2016; Omenetti and Pizarro, 2015). Tregs have been demonstrated to play a significant role in the pathogenesis of DKD (Alikhan et al., 2018; Qin et al., 2021). Tregs treatment improves insulin resistance and ameliorates the development of DKD (Eller et al., 2011; Mosterd et al., 2021).

In diabetes patients with gut microbiota dysbiosis, the intestinal barrier becomes more permeable, resulting in the leakage of lipopolysaccharide (LPS) into the bloodstream and infiltration of bacteria byproducts, further leading to inflammation and exacerbating the development of DKD (Cai et al., 2020; Lin et al., 2022; Ni et al., 2022; Wang P. et al., 2021; Wang Y. et al., 2022). Increased levels of circulating trimethylamine oxide (TMAO), one of the intestinal microbiota metabolites, may also exert pro-inflammatory effects through NLRP3 activation and nuclear NF-κB signals, contributing to renal interstitial fibrosis and dysfunction in DKD (Fang Q. et al., 2021; Mosterd et al., 2021; Wang Y. et al., 2022). And continuous accumulation of intestinal microbiota metabolites, such as PS, TMAO, IS and PCS, could also activate complement C5 and stimulate the immune system, which could result in overproduction of inflammatory factors and renal damage in DKD (Fang Y. et al., 2021; Wang P. et al., 2021). On the other hand, the decrease of several beneficial SCFAs producing bacteria, especially butyrate-producing bacteria, exacerbate the pro-inflammatory environment (Lv et al., 2022; Sabatino et al., 2017; Zaky et al., 2021). Because SCFAs have shown positive effects on DKD through inhibition of inflammation and immune response (Cheng et al., 2022; Feng et al., 2018; Huang et al., 2020; Lv et al., 2022). Previous research suggested that SCFAs could protect the gut barrier from the disruption of LPS through inhibiting NLRP3 inflammasome (Feng et al., 2018). And SCFAs can alleviate systemic inflammation in DKD by activating through inhibiting histone deacetylase (HDAC) (Chi et al., 2021; Lv et al., 2022; Rooks and Garrett, 2016). Recent study has suggested that the elevated complement C5 activation induce declined gut microbiota diversity and decreased SCFAs production, thus promoting renal inflammation and dysfunction in T2DM (Li et al., 2021).

Taken together, these findings suggest that a close relationship might exist between the gut microbiota and inflammation and immunity in DKD, which may pave a new way for DKD treatment (Figure 1).

### 3.4 Gut microbiota dysbiosis and oxidative stress in DKD

Hyperglycemia is the driving force of DM, and oxidative stress caused by hyperglycemia has been regarded as the initial part of renal damage and plays a central role in the progression DKD (Chen W. et al., 2021; Donate-Correa et al., 2015; Samsu, 2021). Oxidative stress is the result of the imbalance between the production of ROS and the antioxidant defense system (Pizzino et al., 2017; van der Pol et al., 2019). Increased ROS causes DNA damage, protein and lipid peroxidation, resulting in irreversible cell damage (van der Pol et al., 2019).

Oxidative stress could induce renal damage in patients with DM directly or indirectly. On the one hand, oxidative stress can cause direct damage to all types of kidney cells, such as podocytes, mesangial cells, endothelial cells and tubule cells (Donate-Correa et al., 2015; Samsu, 2021). As hyperglycemic-induced oxidative stress induces glycoxidation and peroxidation, leading to increased oxidant-derived renal injury in patients with DM (Aghadavod et al., 2016). On the other hand, indirectly, oxidative stress causes renal injury through participating in many pathological pathways of DKD, including RAS activation, inflammation, immunity, and fibrosis (Jha et al., 2016; Kashihara et al., 2010; Samsu, 2021; Sapian et al., 2022). Oxidative stress promotes macrophages to secrete inflammatory cytokines, causing local and systemic inflammation (Samsu, 2021). Oxidative stress can also induce increased Ang-II levels and TGF-β activation, which stimulates mesangial matrix synthesis, glomerulosclerosis, and promotes renal tubulointerstitial fibrosis in DM (Aghadavod et al., 2016; Samsu, 2021).

In fact, numerous studies have shown that gut microbiota dysbiosis can trigger oxidative stress and play a key role in the pathogenesis of DKD (Fang Q. et al., 2021; Mosterd et al., 2021; Ni et al., 2022; Tao et al., 2022). It has been demonstrated that increased circulating levels of typical gut microbiota-derived uremic toxins, including PCS, IS, PS and TMAO, trigger oxidative stress and increase the production of ROS, which activates NLRP3 inflammasome, leading to inflammation in glomerular endothelial cells and exacerbating renal dysfunction of DKD (Chen et al., 2017; Fang Q. et al., 2021; Huang et al., 2017a; Mosterd et al., 2021; Ni et al., 2022). In addition, Huang et al. reported that SCFAs, especially butyrate, ameliorate DKD via GPR43-mediated inhibition of high glucose-induced oxidative stress and NF-κB signaling (Huang et al., 2020). In conclusion, gut microbiota may provide a plausible connection between oxidative stress and DKD (Figure 1).

## 4 Application of gut microbiota-targeted therapies in DKD

**FIGURE 2:**
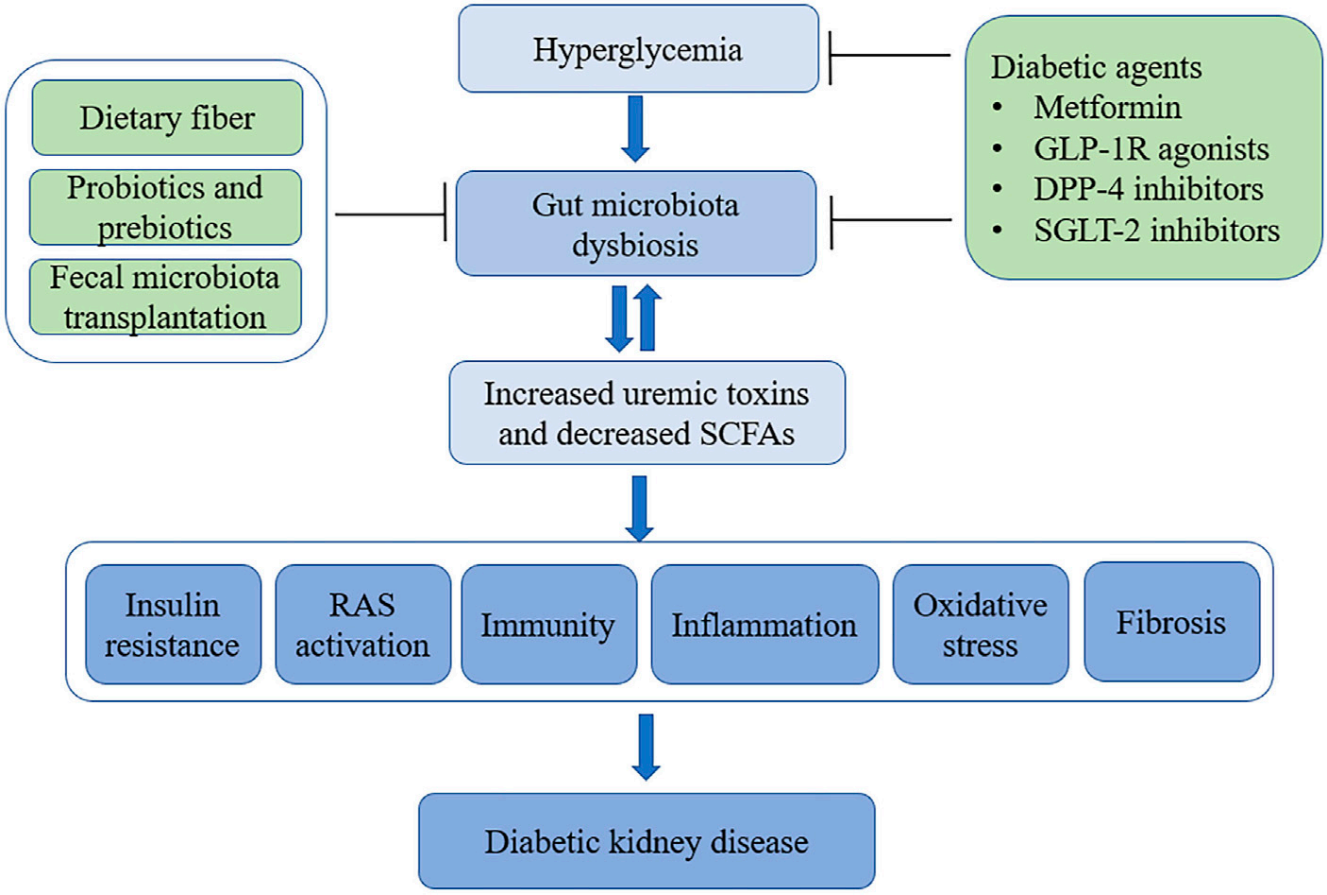
The gut-kidney axis in the pathogenesis of DKD and application of gut microbiota-targeted therapies. In patients with diabetes, hyperglycemia promotes gut microbiota dysbiosis, which contributes to the development of DKD. Gut microbiota-targeted therapies including dietary fiber, supplementation with probiotics or prebiotics, fecal microbiota transplantation and diabetic agents that modulate the gut microbiota, such as metformin, GLP-1R agonists, DPP-4 inhibitors, and SGLT-2 inhibitors. (DKD, diabetic kidney disease; SCFAs, short-chain fatty acids; RAS, renal renin-angiotensin system; GLP-1, glucagon-like peptide-1 receptor; DPP-4, dipeptidyl peptidase-4; SGLT-2, sodium-glucose cotransporter 2).

Considering the critical role of gut microbiota dysbiosis plays in the progression of DKD, potential therapeutic strategies that target the gut microbiota are already being investigated for the treatment of DKD (Figure 2).

### 4.1 Diet

Diet is increasingly recognized to be the main exogenous factor that influence the composition of gut microbiome (Li et al., 2020; Zaky et al., 2021). High-fat and high-fructose diet increase uremic toxins and the proportion of an LPS-containing microbiota in the gut, and induce insulin resistance (Cani et al., 2007; Yang et al., 2021). Conversely, high-fiber diet increases the production of SCFAs and alleviates systemic inflammation (Lv et al., 2022; Zhao et al., 2018). Previous studies have revealed that the supplementation of dietary fiber reduced serum levels of uremic toxins and proinflammatory cytokines, and reversed kidney injuries in CKD (Chiavaroli et al., 2015; Felizardo et al., 2019; Zhong et al., 2021). Similarly, recent studies indicated that dietary fiber ameliorates diabetes-related dysbiosis and protect against DKD through promoting SCFA-producing bacteria, which are able to attenuate inflammatory and oxidative stress (Drake et al., 2022; Li et al., 2020; Lv et al., 2022).

Recently, intermittent fasting or time-restricted feeding has also been found to play a protective role in DKD via improving the gut microbiota composition (Fang Y. et al., 2021; Yang et al., 2021). β-hydroxybutyrate (β-HB), the most important ketone body induced by time-restricted feeding, may mitigate podocyte senescence and injury in diabetic mice by attenuating oxidative stress (Fang Y. et al., 2021).

Taken together, these findings suggest that follow a high-fiber diet, intermittent fasting or time-restricted feeding provide novel approaches for treatment of DKD.

### 4.2 Probiotics and prebiotics

Probiotics are live microbial that are indispensable elements for human health. Probiotics synthesize various vitamins and improve the intestinal microbial balance in host. Prebiotics are fermented food components that regulate the composition of gut microbiota and benefit human health (Aydin et al., 2023; Ma et al., 2019). The use of probiotics and prebiotics have been widely investigated on various disease condition. Previous study has revealed that probiotics supplementation could alleviate insulin resistance and affect glucose metabolism in gestational diabetes mellitus (GDM) (Dolatkhah et al., 2015). In type 1 diabetes mellitus (T1DM), Ho et al. found that prebiotic administration improve glycaemic control through positive changes in gut microbiota, gut permeability and inflammation (Ho et al., 2016). Numerous studies have also shown that the supplementation with probiotics or prebiotics can reduce the levels of uremic toxins and blood glucose, restructure gut microbiota, attenuate oxidative stress and inflammation in CKD (Lv et al., 2022; Ramezani et al., 2016; Yang et al., 2021; Yang et al., 2018). Recent study confirmed that oral administration of probiotic exerts anti-inflammatory effects in AKI by increasing the level of SCFAs and alleviates chronic renal interstitial fibrosis (Zhu et al., 2021). Randomized controlled trials (RCTs) have shown that, compared with conventional soy milk, probiotic soy milk consumption could reduce serum IL-18, improve kidney function, improve antioxidant factors and enzymes in type 2 DKD patients (Abbasi et al., 2017; Miraghajani et al., 2019; Miraghajani et al., 2017). In addition, a recent meta-analysis revealed that probiotics significantly improved glucolipid metabolism, alleviated renal impairment, ameliorated inflammation and oxidative stress in patients with DKD (Dai et al., 2022).

Overall, probiotics and prebiotics appear to be a safe and low-cost potential treatment for DKD, and more RCTS with high quality are needed to further clarify the therapeutic effects in the future.

### 4.3 Fecal microbiota transplantation

Fecal microbiota transplantation (FMT) is a rapidly growing method to reconstitute the recipient’s dysbiosis of gut microbiome through fecal transplant from healthy donors (Gulati et al., 2023; Wang et al., 2019). FMT from lean donors has been indicated as a potential treatment in obese patients with metabolic syndrome, with a beneficial modification in intestinal microbiota composition and an improvement in insulin sensitivity (Kootte et al., 2017; Vrieze et al., 2012). Current evidence has regarded FMT as a safety and potential therapeutic method for a series of chronic diseases associated with gut microbiota alterations, including inflammatory and immune diseases (Danne et al., 2021; Ni et al., 2022; Wang et al., 2019; Wang et al., 2018). However, direct evidence of FMT in DKD is rare. Hu et al. for the first time indicated that FMT alleviates tubulointerstitial injury in diabetic rats through mediating the dysregulation of cholesterol homeostasis (Hu et al., 2020). In a recent preclinical mice model of DKD, resembling human DKD alterations, researchers found that FMT prevents body weight gain, decreases albuminuria, reduces local inflammation in intestinal and ameliorates insulin resistance, providing new evidence for the role of FMT in diabetic patients (Bastos et al., 2022). In addition, another recent study conducted by Shang et al. also demonstrated that, compared with canagliflozin treatment, FMT could more obviously decrease the blood glucose and alleviates the pathological damage in DKD mice by affecting gut microbiota (Shang et al., 2022).

In conclusion, healthy FMT can alter gut microbiome and paly a protective role in the development of DKD. However, the long-term outcomes of FMT still need to be elucidated (Yang et al., 2021). In the future, more prospective studies with high-quality are urgent to provide long-term safety and effectiveness data of FMT for clinical application.

### 4.4 Diabetic agents

#### 4.4.1 Metformin

Metformin has been widely regarded as the first-line antidiabetic drug with safety and efficacy (Foretz et al., 2019; Sun et al., 2018). Previous studies have suggested that metformin exerts protective effect in DKD through inhibiting oxidative stress, attenuating inflammation and tubulo-interstitial damage (Kawanami et al., 2020). Increasing studies have reported that metformin improves glucose homeostasis in DKD by increasing the levels of SCFA-producing gut microbiota (de la Cuesta-Zuluaga et al., 2017; Lee et al., 2021; Sun et al., 2018; Vallianou et al., 2019; Wu et al., 2017). Metformin changes the gut microbiome in the upper small intestine and enhances the secretion of the glucose-lowering gut incretin hormone glucagon-like peptide 1 (GLP-1) in gastrointestinal tract (Bauer et al., 2018; Foretz et al., 2019; Lee et al., 2021). In addition, metformin could also strengthen intestinal permeability and regulate the BAs circulation through interacting with the gut microbiota (Lee et al., 2021; Sansome et al., 2020; Sun et al., 2018). However, metformin should be cautiously administered to patients with renal failure, because metformin accumulates and results in metformin-associated lactic acidosis (MALA) (Kawanami et al., 2020; Rhee and Kalantar-Zadeh, 2017).

#### 4.4.2 GLP-1 receptor agonists

GLP-1 is a gut incretin peptide hormone secreted by intestinal L-cells after food intake, which acts by binding to the GLP-1 receptor (GLP-1R), stimulating insulin secretion. And suppressing glucagon secretion (Madsen et al., 2019; Wang J. et al., 2021). The level of GLP-1 is decreased in patients with diabetes, and GLP-1R agonists can improve the function of GLP-1 and exert beneficial effects on kidney function (Ni et al., 2022; Wang J. et al., 2021). Previous study has demonstrated that liraglutide, one of the GLP-1R agonists, could regulate the intestinal microbiota and immunity to improve insulin secretion in diet-induced dysmetabolic mice (Charpentier et al., 2021). And liraglutide was proved to change the composition of the gut microbiome, regulate glucolipid metabolism and attenuate intestinal inflammation (Kato et al., 2021; Madsen et al., 2019). Recent studies also suggested that liraglutide modulates gut microbiome and exerts renoprotective effects in the treatment of DKD (Ni et al., 2022; Wang J. et al., 2021). Further studies are urgent to explore the effect of other GLP-1R agonists (lixisenatide, dulaglutide and exenatide) on gut microbiota and DKD.

#### 4.4.3 DPP-4 inhibitors

Dipeptidyl peptidase-4 (DPP-4) is the degradation agent of GLP-1 and glucagon-like peptide-2 (GLP-2), which are both glucagon-derived peptides and secreted from intestinal endocrine L cells (Ning et al., 2020; Sun et al., 2020). The glucose lowering effect of GLP-1 has been widely proved in patients with DM (Sun et al., 2020). GLP-2 improves gut barrier function, enhances intestinal blood flow, ameliorates inflammation, and repairs damaged intestinal epithelium (Abdalqadir and Adeli, 2022; Yan et al., 2016).

DPP-4 inhibitors can increase the level of GLP-1 and GLP-2 by inhibiting DPP-4 (Ning et al., 2020; Yan et al., 2016). Studies have confirmed that DPP-4 inhibitors exhibit renoprotective effects via inhibiting inflammation and oxidative stress, preventing podocyte injury, and delaying glomerulosclerosis in DKD (Kawanami et al., 2021; Kubo et al., 2020; Mima, 2022). Sitagliptin, a DPP-4 inhibitor, was reported to regulate the dysbiosis of gut microbiota in a rat model of diabetes, and the potential beneficial effect may be related to GLP-2 (Yan et al., 2016). In diabetic fatty rats model, sitagliptin has been found to regulate the gut microbiota and increase the abundance of *Lactobacillus*, which exhibits antidiabetic effects by stimulating incretin hormones secretion (Zhang et al., 2019). And another DPP-4 inhibitor vildagliptin was found to increase SCFA-producing bacteria and alleviate insulin resistance in diabetic rats (Zhang et al., 2017).

However, in a 12-week RCT conducted in patients with T2DM, researchers found that neither the GLP-1R agonist liraglutide nor the DPP-4 inhibitor sitagliptin changes the fecal microbiota composition (Smits et al., 2021). Taken together, the potential renoprotection mechanisms between DPP-4 inhibitors and intestinal microbiota in DKD remains to be elucidated.

#### 4.4.4 SGLT-2 inhibitors

Sodium-glucose cotransporter-2 (SGLT-2) inhibitors have been demonstrated to lower blood glucose by inhibiting SGLT-2 absorption of glucose in the proximal tubule of the kidney (Polidori et al., 2013; Toto, 2017). SGLT-2 inhibitors could reduce intestinal glucose absorption and increase GLP-1 secretion via suppressing sodium-glucose cotransporter-1(SGLT-1), which is highly expressed in the intestinal mucosa (Polidori et al., 2013; Lehmann and Hornby, 2016).

SGLT-2 inhibitors have been widely used in clinical to improve renal outcomes in patients with T2DM (Perkovic et al., 2019; Mima, 2022). Apart from the recognized renal protective mechanism of inhibiting oxidative stress, inflammation and fibrosis, reducing blood glucose and blood pressure (Kashihara et al., 2020; Wang J. et al., 2021), SGLT-2 inhibitors were also reported to alter the intestinal microbiota in type 2 diabetic rats and patients (Deng L. et al., 2022; Deng X. et al., 2022; Lee et al., 2018; Wang X. et al., 2022; Yang M. et al., 2020). Studies have shown that SGLT-2 inhibitors improve vascular dysfunction and maintain mitochondrial homeostasis, which may be associated with alteration of gut microbiota composition (Deng X. et al., 2022; Lee et al., 2018; Wang X. et al., 2022). Canagliflozin have been demonstrated to reduce the accumulation of uremic toxins and increase SCFAs-production microbiota in a CKD mouse model (Mishima et al., 2018). In addition, a recent study has confirmed that empagliflozin attenuates DKD via altering the gut microbiota, with reduced LPS-producing bacteria and increased SCFA-producing bacteria (Deng L. et al., 2022). However, in a 12-week double-blind RCT in patients with T2DM, neither treatment with dapagliflozin or gliclazide altered the fecal microbiome (van Bommel et al., 2020).

In summary, the renoprotective effect of SGLT-2 inhibitors is beyond doubt, its association with gut microbes in DKD treatment is worthy of detailed exploration.

In addition to the diabetes agents mentioned above, accumulating studies have explored the association between the traditional Chinese medicine and gut microbiota modulation in the treatment of DKD, such as resveratrol, cordyceps cicadae polysaccharides, San-Huang-Yi-Shen Capsule, and Shenyan Kangfu tablet (Cai et al., 2020; Chen Q. et al., 2021; Su et al., 2021; Yang J. et al., 2020). In future, more novel diabetes agents that targeting the gut gut-kidney axis remains to be explored.

## 5 Conclusion and outlook

DKD is a major diabetic complication in patients with DM. The gut microbiota dysbiosis has been confirmed to participate in the progression of DKD, involving insulin resistance, RAS activation, oxidative stress, inflammation and immunity. Gut microbiota-targeted therapies include following a high-fiber diet, supplementation with probiotics or prebiotics, fecal microbiota transplantation and diabetic agents that modulate the gut microbiota, such as metformin, GLP-1R agonists, DPP-4 inhibitors, and SGLT-2 inhibitors. These studies suggest that modulation of the gut-kidney axis is a promising therapeutic target for controlling the progression of DKD. Therapeutic interventions to alter gut microbiota dysbiosis could be applied to ameliorate the kidney damage of DKD. Future efforts should aim to fully understand the potential renoprotection mechanisms between intestinal microbiota and DKD, and it is worthy of further investigation in humans.
